# Linkages between men’s wealth status and the ideal number of children: A trend and multilevel analysis of survey data in Nigeria

**DOI:** 10.1371/journal.pgph.0001036

**Published:** 2023-03-27

**Authors:** Adewole G. Ololade, Blessing I. Babalola, Kehinde O. Omotoso, Oyeyemi O. Oyelade, Elhakim A. Ibrahim

**Affiliations:** 1 National Centre for Technology Management, Obafemi Awolowo University, Ile-Ife, Nigeria; 2 Federal University of Oye Ekiti, Oye Ekiti, Ekiti State, Nigeria; 3 Department of Nursing Science, Obafemi Awolowo University, Ile-Ife, Nigeria; 4 The University of Texas at San Antonio, San Antonio, Texas, United States of America; Northwestern University Feinberg School of Medicine, UNITED STATES

## Abstract

Most African societies practice a patriarchal family system that endows a man with authority and dominance in the family and society with a defined role of being the breadwinner of the home. A man is expected to have a great influence in determining the ideal number of children in the family and take a domineering role in decision-making, especially those related to household resource allocation. Therefore, this study examines the relationship between men’s wealth status and an ideal number of children. The study used secondary data from the National Demographic Health Survey (NDHS) from 2003 to 2018. The objectives were achieved using descriptive and inferential statistics, including frequency, mean, ANOVA, and multilevel analysis techniques. Wealth status significantly influenced the ideal number of children considering the crude and adjusted regression analysis. After adjusting for individual-level and contextual factors, the odd ratio of ideal number of children was significantly lower among men in the richest categories of the wealth index. Moreover, men with two wives and above, uneducated men, Northern residents, men living in high community family norms, low community family planning, high community poverty, and low community level of education desired a high number of children. The analyses suggest the need for a consideration of community structures to provide lucrative employment for men and would experience an appreciable fertility decline in line with the objectives and targets stated in Nigeria’s population policies and programmes.

## Introduction

Men and women usually develop an ideal family size from their young age [1], which they intend to achieve in their reproductive life or family life. Ideal family size is expected to guide their reproductive life. Unfortunately, it may not be achieved as intended [2], and it could change over time [1]. Evidence suggests that the achieved number of children in Nigerian families will continue to increase if all socio-demographic factors that encourage fertility are in place [3].

In most African countries, specifically in Nigeria, families are mostly patriarchal [4], which confers authority on men as husbands and fathers [5], and men are expected to be the breadwinner of the home [6], even if their partners are working they are expected to work more time and earn more to take care of their home while their wives are expected to care of their children and work part-time [7]. Therefore, the man is expected to have a greatly influence in determining the ideal number of children in the family as they have been reported to take a domineering role in decision-making [8]. It was reported in a study that paternal occupation influenced the achieved number of children but not the ideal number of children in Nigeria. It was further stated that unemployed families, those engaging in agriculture, and the poorest were reported to have a higher ideal number of children compared to their more economically buoyant counterparts and that paternal occupation did not significantly influence an ideal number of children [3]. Despite this, the knowledge gap remains with respect to men’s wealth status as it influences an ideal number of children in Nigeria.

In developed countries, factors such as prevailing norms and wealth status have been reported to influence the ideal number of children in a family from a parenting perspective [9]. However, the patriarchal context of Africa, which recognises men as the breadwinner, specifically in Nigeria has not been sufficiently linked with an ideal number of children in literature, leaving out a gap in knowledge about the linkage between men’s wealth status and an ideal number of children in Nigeria. Despite being explained in the literature that prevailing norms per time can lead to changes in the ideal number of children desired over time [9], it leaves out a gap in knowledge about trends in the linkage between the wealth status of men and the ideal number of children over time. It was revealed that due to certain complexities around the concept of wealth, cross-sectional studies do not establish the linkages between wealth status and fertility [10], but this could be captured longitudinally. Yet longitudinal studies that examine these relationships are insufficient in Nigeria. This poses the puzzling question of whether there had been an intergenerational change in men’s ideal number of children as their wealth status changed over the years or whether the ideal number of children is the same over time in Nigeria.

In addition, it is an adage that “the more the children in a family, the more their poverty”, implying that there are community norms or factors that guide family size and wealth status. It was reported in a study done in the Philippines that there is a regressive and negative impact of the increased number of children on family welfare and a strong impact on poverty occurrence [11] which corroborated the adage. However, it has not been sufficiently established in literature the extent to which community factors influenced men’s wealth status and fertility desire in Nigeria.

### Research Question

- Are there wealth differentials in men’s fertility desire between 2003 and 2018
- What other factors influence men’s fertility desire across the time periods?

## Materials and methods

Data used in this analysis was sourced from four consecutive waves of the Nigeria Demographic and Health Surveys (NDHS), including 2003, 2008, 2013, and 2018 surveys [12–15]. Three questionnaires were used in the 2003, 2008, 2013 and 2018 NDHS: the Household Questionnaire, the Women’s Questionnaire, and the Men’s Questionnaire. However, for this study, the Men’s Questionnaire was analysed. The 2003 survey was the second survey, while the most recent survey in the 2018 survey. The NDHS has repeated cross-sectional surveys collected quinquennially by the National Population Commission, with new samples drawn. The respective men’s sample sizes for 2003, 2008, 2013, and 2018 are 2346, 15486, 17359, and 13311. The survey covers the entire population residing in non-institutional dwelling units in the country. Each sample follows a multi-stage stratified design such that each sample is nationally representative in rural and urban areas within any year. Population weights are available in the surveys for both households and individuals to account for the different survey designs among the datasets. NDHS gathers a rich array of demographic and socio-economic information on households and individuals across the country’s six geopolitical zones. Survey questions relate to social services, socio-demographic information, labour markets, health, and health care information, amongst others. Pertinent to this study, the surveys contain information on population and fertility indicator estimates at the national, zonal, and state levels. The sample design allowed for specific indicators to be calculated for each of the six zones, 36 states, and the Federal Capital Territory, Abuja. The survey used as a sampling frame the list of enumeration areas (EAs) prepared for the 2006 Population Census of the Federal Republic of Nigeria and provided by the National Population Commission (NPC).

### Variables definition and measurements

The outcome variable was an ideal number of children. The main independent variable was the wealth index. In the survey, several questions were asked on the ownership of several assets. Thus, the calculation of the wealth index was based on questions relating to ownership of household assets and certain goods, housing type and features, source of drinking water, sanitation amenities, and other features that are associated with the household’s socioeconomic status. The assets were individually assigned a weight (factor score) which was computed from principal component analysis (PCA). Subsequently, the estimated asset scores were standardised. Each asset was allocated a score and a total score was estimated for each household [14] that had already generated the score for each respondent as contained in the individual-level data. The aggregate score was subsequently categorised into five categories poorest, poorer, middle, richer, and richest.

Other pertinent explanatory variables at the individual and community levels include age, religion, knowledge of modern contraceptives, number of spouses, ethnicity, education, age at marriage, occupation region, place of residence, community poverty level (average proportion of households in the bottom two wealth quintiles), community family planning (defined as the percentage of men that know modern contraceptive methods within the community, categorised into low, middle, and high.), community-level of education (defined as the percentage of men that have at least secondary level of education within the categorised categorized into low, middle, and high.), community ideal number of children (defined as the mean number of ideal children per man in the community). The choice of these variables is informed by the theory propounded by Bongaarts [16–19] and other several theoretical propositions which hypothesized a positive relationship between wealth and an ideal number of children [19–24]. Bongaarts advocates that some socio-economic, cultural, and environmental factors influence decisions on fertility and an ideal number of children to have by men or in a family setting [16]. Essentially, these factors exert influence through proximate determinants which directly affect fertility. The proximate determinants comprise the use of contraceptives and the proportions that are married, amongst others. A considerable body of work has also demonstrated that clear positive relationships exist between wealth and fertility for both men and women, especially in societies with a high level of fertility and high inclinations towards having many children. The main argument is that individuals utilise their resources to increase reproductive succes, sometimes, equivalent to having a relatively high number of children. Stulp investigates the relationship between wealth and fertility in modern populations and shows a positive relationship between wealth and giving birth to many children in natural fertility populations, which are usually considered ideal models within a wealthy context [25]. Nevertheless, Vining & Peruse find that the relationship becomes negative during the fertility transition [26, 27]. The veracity of this finding was, however challenged by Stulp who opines that even in post-transition societies, fertility and having the number of children considered as the perfect model are still highly correlated with wealth when longitudinal datasets are appropriately analysed, though these relationships might be complex [25] and stronger for men than for women and may vary across different ethnic groups within complex societies, implying that it is important not to treat modern complex societies as an undifferentiated whole but to address and use variation within societies when testing these hypotheses. Thus, this study will contribute to the existing knowledge base by testing the hypothesis that men’s wealth is positively related to an ideal number of children by using nationally representative datasets.

### Statistical analysis

The relationship between both the individual and community determinants across an ideal number of children being a count variable was analysed using ANOVA and T-test (Tables 1 and 3). ANOVA was done to test if there was an association between the explanatory variables with three categories and the outcome variable, while T-test was done on variables with just two categories and the outcome variables. Moreover, the frequency, mean and standard deviation of the explanatory variables in relationship with the outcome variable, which is the ideal number of children, was reported in the ANOVA and T-test (Tables 1 and 3). Post-hoc test was conducted. Post-hoc pairwise comparisons are commonly performed after significant effects when there are three or more levels of a factor when testing the association using ANOVA. It was performed to show a significant difference in the mean ideal number of children in relation to the independent variables. The pairwise method that was used was Bonferroni. Bonferroni tests both simple and complex selected contrasts. At the multivariate analysis, multilevel Poisson regression was run. To examine how variation was built up from crude and adjusted model, a separate analysis was done examining the relationships between the ideal number of children and poverty level (first column; unadjusted), and the second column shows the relationship between the ideal number of children with poverty level adjusting for an individual- and community-level variables for the four-year analysis. Generalized linear and latent mixed models (GLLAMM, downloadable program, and implementable in Stata version 13.0) were used to conduct all the multilevel analysis. Fixed effects and random effects, which are important concepts in the multilevel analysis, were employed in the interpretation of the results. While fixed effects are used to model associations, random effects are useful in modelling variations [28, 29].

**Table 1:** Mean ideal number of children, according to individual-level characteristics in Nigeria.

Variables	2003			2008			2013			2018		
	N	(SD)	F (ANOVA)	N	(SD)	F (ANOVA)	N	(SD)	F (ANOVA)	N	(SD)	F (ANOVA)
**Wealth index**												
Poorest	368	11.4(9.3)	58.7*	2548	11.1(8.9)	423.75*	2455	12.6(8.2)	735.79*	2122	11.0(7.0)	538.75*
Poorer	318	11.6(9.8)		2421	9.9(8.3)		2849	9.8(7.2)		2285	9.3(6.6)	
Middle	357	9.6(8.9)		2691	8.0(7.4)		3323	8.0(6.6)		2718	7.8(5.8)	
Richer	431	7.4(6.7)		3004	6.2(5.0)		3870	6.5(5.4)		2771	5.9(4.2)	
Richest	518	5.0(3.0)		2903	4.8(3.3)		3957	5.0(3.4)		2629	4.8(3.0)	
**Current age**												
15–24	797	7.2(5.9)	38.8*	4575	6.4(5.1)	170.89*	6293	7.1(5.7)	205.22*	3776	6.7(4.6)	123.09*
25–34	532	8.0(8.0)		4042	7.0(6.7)		4878	7.5(6.1)		3203	6.7(5.0)	
35 and above	663	10.7(9.6)		4950	8.9(8.1)		5283	9.4(7.8)		5546	8.3(6.6)	
**CEB**												
<5 children	1521	7.3(6.5)	183.7*	10322	6.4(5.5)	1245.02*	13468	7.0(5.6)	1803.39*	9477	6.2(4.3)	2184*
5+ children	471	12.8(10.5)		3245	11.0(9.4)		2986	12.4(9.0)		3048	11.4(7.7)	
**Religion**												
Christianity	1155	6.0(4.2)	160.5*	7527	5.5(3.9)	271.39*	8733	5. 2(3.0)	1607.07*	6408	4.9(2.6)	1341.04*
Islam	801	12.1(10.2)		5763	10.3(8.9)		7493	10.6(8.0)		6013	9.8(6.8)	
Others	36	8.4(6.1)		249	8.2(7.6)		156	8.4(5.0)		104	7.2(5.3)	
**Knowledge of modern contraceptives**												
Yes	1862	8.5(8.1)	2.85	1464	9.5(8.1)	121.82*	900	9.8(7.5)	72.93*	593	8.6(5.9)	26.3*
No	130	9.7(5.8)		12103	7.3(6.7)		15554	7.9(6.6)		11932	7.4(5.7)	
**Number of spouses**												
A wife	740	8.9(8.2)	110.23*	6037	7.7(6.8)	783.69*	6689	8.1(6.2)	1320*	6493	7.0(5.0)	1884.07*
2 or more wives	201	16.7(12.4)		1295	14.5(11.3)		1291	15.7(9.9)		1025	15.0(8.6)	
**Ethnicity**												
Hausa	454	14.2(10.9)	155.72*	3560	12.4(9.5)	954.15*	4949	11.7(8.4)	1254.22*	4128	10.7(7.1)	1082.94*
Igbo	375	5.2(2.5)		1843	5.3(3.4)		2144	5.1(2.5)		2113	4.8(2.3)	
Yoruba	324	4.7(2.0)		2299	4.9(2.7)		2339	4.5(1.7)		1766	4.4(2.2)	
Others	839	7.7(6.5)		5841	6.7(5.9)		6978	6.8(5.2)		4512	6.6(4.6)	
**Educational level**												
No education	316	14.4(10.5)	90.80*	2711	12.4(9.6)	666.01*	3076	12.4(8.2)	846.79*	2594	11.4(7.1)	677.36*
Primary	530	9.0(8.1)		2863	8.4(7.3)		2784	8.8(6.7)		1797	7.9(6.1)	
Secondary	897	6.8(5.9)		6057	6.0(4.9)		8055	6.5(5.4)		5959	6.1(4.3)	
Higher	249	6.5(6.5)		1936	5.2(4.3)		2539	5.8(4.6)		2175	5.9(4.6)	
**Age at marriage**												
<18	103	13.1(11.0)	21.32*	573	11.6(9.7)	147.78*	505	11.8(9.0)	187.90*	391	10.2(7.1)	158.13*
18–24	431	11.9(10.6)		3414	10.1(8.6)		3524	10.7(8.0)		2834	9.5(7.1)	
25+	464	8.4(7.7)		36.5	7.3(6.8)		4205	7.8(6.3)		4470	7.0(5.5)	
**Occupation**												
Not working	598	6.5(5.1)	41.4*	2201	5.2(3.5)	271.39*	3467	6.3(5.1)	301.8*	1390	5.4(3.6)	272.45*
Formal	239	6.9(6.6)		2418	6.3(5.4)		2586	6.5(5.9)		2616	6.1(4.8)	
Informal	778	10.9(9.3)		6616	9.2(8.1)		6596	9.7(7.6)		6612	8.8(6.3)	
Manual labour	377	8.2(8.5)		2262	6.7(6.3)		3658	7.3(5.9)		1907	6.4(5.3)	

* = p value < 0.05

Two models were fitted in the multilevel analysis for each year’s data.

Model 1—This model only considers the wealth index as the only explanatory variable in the model

Model 2 –This full model incorporates all variables (wealth index, individual/household, and community variables) into the multilevel analysis.

AIC and BIC are Akaike and Schwarz’s Bayesian information criteria. Models with smaller values of an information criterion are considered preferable.

## Results

### Mean ideal number of children by poverty level and selected characteristics

Table 1 shows the data on the number of men’s ideal number of children in Nigeria by individual variables. Each cell in the table shows the mean, the standard deviation, and the number of observations. The ideal number of children on average was higher among men with two wives or more than those with one wife (16.7 versus 8.9 for 2003, 14.5 versus 7.7 for 2008, 15.7 versus 8.1 for 2013, and 15.0 versus 7.0 for 2018), had five or more children than those with less than five children (12.8 versus 7.3 for 2003, 11.0 versus 7.4 for 2008, 12.4 versus 7.0 for 2013 and 11.4 versus 6.2 for 2018) had knowledge of modern contraceptives than those with no knowledge (9.5 versus 7.3 for 2008, 9.8 versus 7.9 for 2013, and 8.6 versus 7.4 for 2018) except year 2003 where men that had no knowledge about contraceptives has a higher ideal number of children than those with knowledge of modern contraceptives (9.7 versus 8.5 for 2003)Moreover, the mean ideal number of children was highest among men who were in the poorest category in all the round of survey except for year 2003 that has its highest in the poorer category (11.6 for 2003, 11.1 for 2008, 12.6 for 2013, and 11.0 for 2018), aged 35 and above (9.6 for 2003, 8.9 for 2008, 9.4 for 2013 and 8.3 for 2018), were Hausas (14.2 for 2003, 12.4 for 2008, 11.7 for 2013 and 10.7 for 2018), were Muslims (12.1 for 2003, 10.3 for 2008, 10.6 for 2013 and 9.8 for 2018), were below age 18 (13.1 for 2003, 11.6 for 2008, 11.8 for 2013 and 10.2 for 2018), who had no education (14.4 for 2003, 12.4 for 2008 and 2013 and 11.4 for 2018) and working in the informal sector (10.9 for 2003, 9.2 for 2008, 9.7 for 2013 and 8.8 for 2018). All the relationships between individual-level characteristics and the outcome variable were significant at p<0.05 except knowledge of modern contraceptive methods in the year 2003. The Bonferroni post hoc test in Table 2 showed a pairwise relationship between the categories of the variables. The results showed that there was a significant relationship between the paired categories for the four surveys except between the poorest and poorer categories in the year 2003. The table further showed that there was a significant relationship between the different categories of religion in all the round surveys. There was a significant difference in a mean number of children ever born between the uneducated men and primary level of education, secondary and higher level of education. Likewise, there was a significant difference between secondary and higher levels of education in the 2008 and 2013 data set while it was not significant for years 2003 and 2018. The table further shows that there was a significant relationship between the Hausas and other tribes such as the Igbos, Yoruba and others categories in all year round but there was no significant difference between the Igbo and Yoruba in all the rounds except in 2013. There was a significant relationship between the pairwise age group in 2003, 2008 and 2013 while there was no relationship in the paired categories for the year 2018.

**Table 2:** Bonferroni post hoc table of the mean difference of ideal number of children with individual and household characteristics.

Variables	Year 2003				Year 2008				Year 2013				Year 2018			
Wealth Index	Poorest	Poorer	Middle	Richer	Poorest	Poorer	Middle	Richer	Poorest	Poorer	Middle	Richer	Poorest	Poorer	Middle	Richer
**Poorer**	0.170496				-1.23945*				-2.78104*				-1.72088*			
**Middle**	-1.8312*	-2.0017*			-3.12082*	-1.88137*			-4.56821*	-4.56821*			-3.27184*	-1.55095*		
**Richer**	-3.97002*	-4.14051*	-2.13882*		-4.88892*	-3.64947*	-1.76811*		-6.05723*	-3.27619*	-1.48902*		-5.09996*	-3.37908*	-1.82812*	
**Richest**	-6.38201*	-6.55251*	-4.55081*	-2.412*	-6.30912*	-5.06967*	-3.1883*	-1.4202*	-7.56238*	-4.78134*	-2.99417*	-1.50515*	-6.23692*	-4.51604*	-2.96509*	**-**1.13696*
**Religion**	**Christianity**	**Islam**			**Christianity**	**Islam**			**Christianity**	**Islam**			**Christianity**	**Islam**		
**Islam**	5.79027*				4.9225*				5.50396*				4.8453*			
**Traditional**	-.374485	-6.16475*			.651045*	-4.27145*			.488915*	-5.01505*			.0336	-4.8117*		
**Age**	**15–24**	**25–34**			**15–24**	**25–34**			**15–24**	**25–34**			**15–24**	**25–34**		
**25–34**	.828195				.571247*				.362957*				.026509			
**35 years +**	3.54639*	2.7182*			2.46217*	1.89092*			2.35746*	1.9945*			1.61776	1.59125		
**Ethnicity**	**Hausa**	**Igbo**	**Yoruba**		**Hausa**	**Igbo**	**Yoruba**		**Hausa**	**Igbo**	**Yoruba**		**Hausa**	**Igbo**	**Yoruba**	
**Igbo**	-8.98678*				-7.03654*				-6.64369*				-5.94427*			
**Yoruba**	-9.3878*	-.401019			-7.50137*	-.464833			-7.27388*	-.630186*			-6.30781*	-.363541		
**Others**	-6.48304*	2.50374 *	2.90476*		-5.7012*	1.33534*	1.80018*		-4.92705*	1.71664*	2.34683*		-4.11167*	1.8326*	2.19614*	
**Educational level**	**No education**	**Primary**	**Secondary**		**No education**	**Primary**	**Secondary**		**No education**	**Primary**	**Secondary**		**No education**	**Primary**	**Secondary**	
**Primary**	-5.42928*				-3.95656*				-3.60565*				-3.45268*			
**Secondary**	-7.64477*	-2.21549*			-6.35823*	-2.40167*			-5.91373*	-2.30809*			-5.31448*	-1.8618*		
**Higher**	-7.92764*	-2.49836*	-.282873		-7.13742*	-3.18086*	-.779188*		-6.60814*	-3.0025*	-.694411*		-5.45905*	-2.00636*	-.144565	
	**<18**	**18–24**			**<18**	**18–24**			**<18**	**18–24**			**<18**	**18–24**		
**18–24**	-1.17747				-1.59257*				-1.11008*				-.759825			
**25 and above**	-4.74919*	-3.57173*			-4.37451*	-2.78194*			-4.03817*	-2.9281*			-3.19733*	-2.4375*		
**Occupation**	**Not working**	**Formal**	**Informal**		**Not working**	**Formal**	**Informal**		**Not working**	**Formal**	**Informal**		**Not working**	**Formal**	**Informal**	
**Formal**	.414916				1.04779*				.297527				.720497*			
**Informal**	4.40144*	3.98652*			4.02073*	2.97294*			3.49612*	3.19859*			3.44876*	2.72826*		
**Manual labour**	1.72956*	1.31464	-2.67188*		1.45125*	.403463*	-2.56947*		1.02079*	.723267*	-2.47533*		.985711*	.265214	-2.46305*	

The relationship between community-level variables and the ideal number of children is shown in Table 3. The North East region had the highest mean ideal number of children in 2008, 2013, and 2018 while it was the North West region in 2003. Rural dwellers desire more children than urban dwellers. Moreover, the mean ideal number of children was highest among men living in the community with a high community ideal number of children (9.5 for 2003, 8.6 for 2008, 10.0 for 2013, and 9.6 for 2018), low community poverty (11.2 for 2003, 10.2 for 2008, 11.0 for 2013 and 9.6 for 2018), low community level of education (12.5 for 2003, 11.1 for 2008, 10.9 for 2013 and 10.2 for 2018), Low community family planning (9.2 for 2003, 8.3.for 2008), medium community family planning in 2013 (8.2) and high community family planning in 2018 (8.2). All the community-level variables were significant at the bivariate level, with the outcome variable at p = 0.05.

**Table 3:** Mean ideal number of children, according to community-level characteristics in Nigeria.

Variables	2003			2008			2013			2018		
	N	(SD)	F (ANOVA)	N	(SD)	F (ANOVA)	N	(SD)	F (ANOVA)	N	(SD)	F (ANOVA)
**Regions**												
North Central	407	8.0(7.5)	74.01*	2715	6.8(5.8)	569.84*	2731	6.7(4.7)	722.81*	2326	7.1(4.6)	717.16*
North East	297	12.5(11.0)		2386	12.5(10.5)		2497	11.4(8.6)		2275	10.8(6.7)	
North West	362	12.8(9.6)		2452	11.1(3.7)		4062	10.9(8.1)		2531	10.4(7.2)	
South East	259	5.3(2.6)		1366	5.6(3.7)		1606	5.3(2.7)		1733	4.9(2.4)	
South-South	305	7.0(5.2)		2175	5.5(3.9)		2971	5.4(3.0)		1661	5.0(2.3)	
South West	362	4.8(2.0)		2473	4.9(2.8)		2587	4.6(2.2)		1999	4.4(2.2)	
**Place of residence**												
Urban	817	6.6(5.5)	77.17*	4677	5.8(5.2)	527.4*	6793	6.4(5.6)	752.52*	5257	6.1(4.7)	634.09*
Rural	1175	9.8(8.9)		8890	8.6(7.6)		9661	9.2(7.1)		7268	8.6(6.3)	
**Community ideal number of children**												
Low	389	7.4(7.4)	14.83*	3122	6.6(6.1)	117.91*	3645	5.5(4.0)	710.55*	2851	4.9(3.4)	839.11*
Medium	608	7.6(7.4)		4301	6.8(6.2)		5706	7.1(6.0)		3883	6.2(4.7)	
High	995	9.5(8.3)		6144	8.6(7.7)		7103	10.0(7.6)		5791	9.6(6.6)	
**Community poverty**												
Low	545	11.2(9.3)	53.08*	3805	10.2(8.4)	415.59*	4576	11.0(7.8)	822.82*	3408	9.6(6.5)	371.5*
Medium	589	8.5(8.3)		4093	7.4(6.7)		5046	7.1(5.6)		4048	7.6(5.9)	
High	858	6.9(6.3)		5669	6.1(5.5)		6832	6.4(5.6)		5069	6.1(4.7)	
**community-level of education**												
Low	452	12.5(10.6)	75.53*	3487	11.1(9.0)	667.28*	4609	10.9(7.6)	792.51*	3576	10.2(6.9)	600.97*
Middle	628	7.9(6.9)		4144	6.7(5.6)		4841	6.8(5.7)		4001	6.2(4.3)	
High	912	7.2(6.5)		5936	6.1(5.6)		7004	6.5(5.6)		4948	6.6(5.3)	
**Community family planning**												
Low	593	9.2(8.4)	8.19*	3688	8.3(7.5)	36.48*	3805	7.5(5.8)	15.84*	2650	6.6(4.8)	85.93*
Medium	560	7.5(6.7)		4097	7.3(6.5)		5413	8.2(6.9)		4349	7.0(5.5)	
High	839	9.0(8.4)		5782	7.1(6.7)		7236	8.0(6.8)		5526	8.2(6.3)	

* = p value < 0.05

The Table 4 is the post hoc test for the community variables in this study. No significant relationship low ideal number of children and the medium ideal number of children for year 2003 and 2008 while it was significant in the later years. The table further showed a significant relationship between the different categories of community poverty in all four surveys. There was a significant difference between low and medium, low and high community levels of education in all the rounds of survey, with the exception of medium and high community level of education in the year 2003. In year 2008, there was a significant difference in the mean ideal number of children between all the paired categories. Same was observed for year 2013 and 2018, with the exception of South East and South South in both years.

**Table 4:** Bonferroni post hoc table of the mean difference of ideal number of children with community characteristics.

Variables	Year 2003					Year 2008					Year 2013					Year 2018				
**Community ideal number of children**	**Low**	**Middle**				**Low**	**Middle**				**Low**	**Middle**				**Low**	**Middle**			
Medium	.278923					.219971					1.59245*					1.27037*				
High	2.11331*	1.83439*				1.95081 *	1.73084*				4.58004*	2.98758*				4.62098*	3.35061*			
**Community poverty**	**Low**	**Middle**				**Low**	**Middle**				**Low**	**Middle**				**Low**	**Middle**			
Medium	-2.70095*					-2.79446*					-3.96967*					-1.99516*				
High	-4.3177*	-1.61675*				-4.10485*	-1.31039*				-4.6435*	-.673832*				-3.42525*	-1.43009*			
**Community-level of education**	**Low**	**Middle**				**Low**	**Middle**				**Low**	**Middle**				**Low**	**Middle**			
Medium	-4.58152*					-4.44631*					-4.0616*					-4.00861*	.367031*			
High	-5.25862*	-.677105				-5.04745*	-.601146*				-4.38043*	-.318828*				-3.64158*				
**Community family planning**	**Low**	**Middle**				**Low**	**Middle**				**Low**	**Middle**				**Low**	**Middle**			
Medium	-1.67981*					-1.03324*					.783382*					.388415*				
High	-.16619	1.51362*				-1.18052 *	-.147275				.546214*	-.237168				1.57968*	1.19126*			
**Region**	**North Central**	**North East**	**North West**	**South East**	**South South**	**North Central**	**North East**	**North West**	**South East**	**South South**	**North Central**	**North East**	**North West**	**South East**	**South South**	**North Central**	**North East**	**North West**	**South East**	**South South**
**North East**	4.4419*					5.70662*					4.715*					3.71365*				
**North West**	4.71241*	.270512				4.2521*	-1.45452*				4.23723*	-.477764*				3.25427*	-.459374*			
**South East**	-2.73355*	-7.17545*	-7.44596*			-1.17531*	-6.88193*	-5.42741*			-1.32985*	-6.04484*	-5.56708*			-2.20232*	-5.91597*	-5.4566*		
**South South**	-1.34991	-5.7918*	-6.06232*	1.38364		-1.27739*	-6.98401*	-5.52949*	-.102081*		-1.25535*	-5.97034*	-5.49258*	.074498		-2.07391*	-5.78756*	-5.32818*	.128413	
**South West**	-3.27655*	-7.71845*	-7.98896 *	-.543003	-1.92664	-1.91104*	-7.61766*	-6.16314*	-.735731*	-.633649*	-2.02754*	-6.74254*	-6.26477*	-.697697*	-.772195*	-2.74337*	-6.45702*	-5.99765*	-.541049*	-.669462*

Table 5 shows the fixed and random effect of the crude and adjusted odd ratio for the relationship between an ideal number of children and poverty level (net of selected individual and community-level variables).

**Table 5:** Multilevel poisson regression of the association between the poverty level and the ideal number of children among men in Nigeria.

Characteristics	2003		2008		2013		2018	
	OR (95% CI)		OR (95% CI)		OR (95% CI)		OR (95% CI)	
	Crude	Adjusted	Crude	Adjusted	Crude	Adjusted	Crude	Adjusted
**Wealth index**								
Poorest (ref)	1	1	1	1	1	1	1	1
Poorer	0.96	0.97	0.97	1.05*	0.88*	0.94*	0.89*	0.96
	(0.86–1.05)	(0.86–1.10)	(0.93–1.01)	(1.01–1.10)	(0.85–0.90)	(0.91–0.98)	(0.85–0.92)	(0.93–1.00)
Middle	0.79*	0.84*	0.85*	1.01	0.78*	0.93*	0.79*	0.94*
	(0.71–0.88)	(0.72–0.97)	(0.82–0.89)	(0.96–1.07)	(0.75–0.81)	0.88–0.97	(0.76–0.82)	(0.89–0.98)
Richer	0.66*	0.80*	0.73*	0.94	0.69*	0.89*	0.71*	0.91*
	(0.59–0.74)	0.67–0.95	(0.70–0.77)	(0.88–1.00)	0.66–0.72	(0.84–0.94)	(0.68–0.74)	(0.86–0.96)
Richest	0.55*	0.75*	0.65*	0.91*	0.60*	0.82*	0.62*	0.87*
	(0.49–0.61)	(0.62–0.92)	(0.61–0.68)	(0.84–0.99)	(0.58–0.63)	(0.76–0.87)	(0.59–0.65)	(0.82–0.92)
**Current age**								
15–24		1		1		1		1
25–34		0.94		0.99		1.02		1.12*
		(0.78–1.15)		(0.93–1.06)		(0.97–1.08)		(1.05–1.20)
35 and above		0.93		1.02		1.06		1.17*
		(0.76–1.14)		(0.95–1.09)		(1.00–1.12)		(1.09–1.26)
**CEB**								
<5 children		1		1		1		1
5+ children		1.21*		1.24*		1.24*		1.30*
		(1.10–1.34)		(1.20–1.28)		(1.20–1.28)		(1.27–1.34)
**Religion**								
Christianity		1		1		1		1
Islam		1.16		1.23*		1.34*		1.20*
		(1.00–1.35)		(1.17–1.30)		(1.29–1.40)		(1.16–1.25)
Others		0.93		1.08*		1.03		0.95*
		(0.82–1.05)		(1.02–1.13)		(0.98–1.08)		(0.91–0.99)
**Knowledge of modern contraceptives**								
Yes		1		1		1		1
No		1.08		1.21*		0.98		0.98
		(0.91–1.28)		(1.15–1.27)		(0.92–1.04)		(0.91–1.06)
**Number of spouses**								
A wife		1		1		1		1
2 or more wives		1.42*		1.38*		1.32*		1.37*
		(1.29–1.56)		(1.33–1.43)		(1.28–1.36)		(1.33–1.41)
**Ethnicity**								
Hausa		1		1		1		1
Igbo		0.64*		0.76*		0.87*		0.89*
		(0.51–0.82)		(0.68–0.84)		(0.81–0.97)		(0.82–0.97)
Yoruba		0.61*		0.72*		0.76*		0.82*
		(0.49–0.76)		(0.66–0.79)		(0.71–0.82)		(0.76–0.87)
Others		0.74*		0.82*		0.90*		0.94*
		(0.64–0.87)		(0.77–0.87)		(0.86–0.94)		(0.90–0.98)
**Educational level**								
No education		1		1		1		1
Primary		0.90		0.99		1.00		0.97
		(0.80–1.00)		(0.95–1.03)		(0.96–1.04)		(0.94–1.01)
Secondary		0.84*		0.90*		0.96*		0.94*
		(0.74–0.96)		(0.86–0.95)		(0.93–1.00)		(0.91–0.98)
Higher		0.90		0.82*		0.91*		0.93*
		(0.75–1.09)		(0.77–0.87)		(0.86–0.95)		(0.89–0.98)
**Age at marriage**								
<18		1		1		1		1
18–24		1.08		0.98		0.97		1.03
		(0.95–1.22)		(0.93–1.03)		(0.93–1.02)		(0.97–1.08)
25+		1.06		0.94*		0.91*		1.00
		(0.93–1.22)		(0.89–1.00)		(0.86–0.95)		(0.95–1.05)
**Occupation**								
Not working		1		1		1		1
Formal		0.92		0.99		1.11		1.01
		(0.74–1.09)		(0.87–1.13)		(0.98–1.26)		(0.90–1.13)
Informal		0.96		1.04		1.12*		1.06
		(0.79–1.16)		(0.91–1.17)		(1.00–1.27)		(0.95–1.18)
Manual labour		0.96		1.04		1.10		1.05
		(0.78–1.18)		(0.91–1.18)		(0.97–1.24)		(0.94–1.18)
**Regions**								
North Central		1		1		1		1
North East		1.35*		1.22*		1.28*		1.19*
		(1.17–1.56)		(1.13–1.31)		(1.21–1.35)		(1.13–1.24)
North West		1.11		1.17*		1.09*		1.16*
		(0.91–1.28)		(1.03–1.21)		(1.02–1.15)		(1.10–1.23)
South East		1.08		1.05		0.99		0.93
		(0.85–1.36)		(0.94–1.18)		(0.90–1.08)		(0.86–1.01)
South-South		1.16		0.98		1.04		0.98
		(0.99–1.36)		(0.91–1.06)		(0.98–1.10)		(0.93–1.04)
South West		0.98		0.88*		0.93*		0.87*
		(0.80–1.20)		0.81–0.96		(0.87–0.99)		(0.82–0.93)
**Place of residence**								
Urban		1		1		1		1
Rural		1.04		1.06*		1.00		0.98
		(0.94–1.18)		(1.02–1.14)		(0.96–1.04)		(0.94–1.01)
**Community ideal number of children**								
Low		1		1		1		1
Medium		1.18*		1.08*		1.10*		1.16*
		(1.04–1.34)		(1.02–1.15)		(1.05–1.16)		(1.11–1.21)
High		1.33*		1.25*		1.22*		1.31*
		(1.14–1.56)		(1.17–1.34)		(1.15–1.30)		(1.24–1.38)
**Community poverty**								
Low		1		1		1		1
Medium		1.01		0.97		0.93*		1.01
		(0.89–1.16)		(0.92–1.04)		(0.88–0.98)		(0.97–1.06)
High		1.06		0.96		0.91*		0.98
		(0.88–1.27)		(0.88–1.05)		(0.84–0.99)		(0.92–1.04)
**Community-level of education**								
Low		1		1		1		1
Middle		0.95		0.98		1.04		0.92*
		(0.83–1.08)		(0.92–1.05)		(0.99–1.10)		(0.89–0.96)
High		0.97		1.03		1.03		0.93*
		(0.82–1.15)		(0.94–1.23)		(0.96–1.11)		(0.88–0.98)
**Community family planning**								
Low		1		1		1		1
Middle		0.79*		0.95		0.98		0.96
		(0.70–0.91)		(0.90–1.01)		(0.94–1.03)		(0.92–1.00)
High		0.86		0.87*		0.95		0.93*
		(0.72–1.01)		(0.81–0.94)		(0.89–1.01)		(0.88–0.98)
**Random effects**								
**Community-level**								
Variance (SE)	0.11(0.01)	0.02(0.01)	0.13(0.01)	0.05(0.00)	0.10(0.01)	0.02(0.00)	0.10(0.01)	0.02(0.00)
VPC = ICC (%)	3.2	0.6	3.8	1.5	2.9	0.6	2.9	0.6
Explained variation (PCV) %	Reference	81.8	Reference	61.5	Reference	80.0	Reference	80.0
**Individual-level**								
Variance (SE)	0.20(0.01)	0.19(0.01)	0.20(0.00)	0.17(0.00)	0.14(0.00)	0.12(0.00)	0.12(0.00)	0.08(0.00)
Explained variation (PCV) %	Reference	5.0	Reference	15.0	Reference	14.3	Reference	33.3
Log-likelihood	-5608.46	-2721.039	-37833.18	-20888.38	-44068.99	-21425.42	-33206	-19147.16
**Model fit statistics**								
AIC	11230.93	5520.077	75680.36	41854.77	88151.98	42928.83	66426.01	38372.32
BIC	11270.1	5709.108	75732.96	42123.55	88205.94	43200.78	66478.06	38642.39

*p<0.05, SE = standard error, VPC = variance partition coefficient, ICC = intra-class correlation coefficient, PCV = proportional change in variance, OR = Odd ratio

The results presented in the crude model of Table 5, which contains only the wealth index variable, indicated a significant variation in the ideal number of children with variances ranging from 0.00 to 0.20 across individual levels and variances ranging from 0.00 to 0.13 across communities; also presented for the adjusted model with variances ranging from 0.00 to 0.17 across individual levels, and variances ranging from 0.00 to 0.05 across communities levels; thereby justifying the use of multilevel modelling in the study. The VPC for 2008 (3.8%) crude model was larger than the VPC in the other years (3.2 for 2003; 2.9 for 2013 and 2018) This suggests that intra-community variations associated with the ideal number of children in 2008 were larger than the observed variations associated with the ideal number of children in other years of observation. The proportional change in variance (PCV) in the adjusted model indicated that 81.8% (2003), 61.5% (2008), and 80.0% (2013 and 2018) of the variance associated with the ideal number of children across communities was explained by the explanatory variables. The estimated PCV shows that more variation was explained by the variables in 2003 compared to other years.

Wealth indexes were significantly associated with an ideal number of children in crude and adjusted models (p<0.05). After adjusting for individual-level and contextual factors, the odd ratio of an ideal number of children was significantly lower among men in the richest categories of wealth index (Odd ratio: 0.75, 95% CI: 0.62–0.92 for 2003; 0.91, 95% CI: 0.84–0.99 for 2008; 0.82, 95% CI: 0.76–0.87 for 2013; 0.87, 95% CI: 0.82–0.92 for 2018 p<0.05) compared to those who were in the poorest categories. Also, Table 5 reveals a higher likelihood of ideal number of children among men in 2003 (Odd ratio: 1.21, 95% CI: 1.10–1.34), 2008 and 2013 (Odd ratio: 1.24, 95% CI: 1.20–1.28) and 2018 (Odd ratio: 1.30, 95% CI: 1.27–1.34), these relationships were statistically significant.

Men with two or more wives are more likely to have a higher ideal number of children than men with a wife, the educated men are likely to have a low ideal number of children than uneducated men. Men in the Southwest region have the lowest likelihood than men in the other region. The community’s ideal number of children was significant in 2008, 2013, and 2018 except in 2003. The odd ratio of an ideal number of children was significantly higher among men living in the high community ideal number of children than their counterparts (2003Odd ratio: 1.33, 95% CI: 1.14–1.56, 2008Odd ratio: 1.25, 95% CI: 1.17–1.34, 2013 (Odd ratio: 1.22, 95% CI: 1.15–1.30) and 2018 (Odd ratio: 1.31, 95% CI: 1.24–1.38).

The crude and the adjusted model for 2003 AIC value was the smallest, and it is preferable, next to it is 2018, both crude and the adjusted model.

## Discussion and conclusion

This study is guided by Bongaart’s theory which shows economic factors as one of the indirect determinants of fertility behaviour [16]. This study examined the linkages between men’s wealth status, community factors, and an ideal number of children between 2003 and 2018 NDHS data. The relationship between men’s wealth status and an ideal number of children between 2003 and 2018 was significant considering the crude and the adjusted odd ratio. The richest were less likely to have the high ideal number of children ever born compared to men in the poorest category. This is in tandem with the findings by Vining & Peruse [26, 27] that the relationship between wealth and fertility becomes negative during the fertility transition. Therefore, it does not correspond with Stulp’s [25] opinions.

Also, the result shows not many intergenerational differences over the years. Still, as men are moving away from the poverty line, the odd ratio value keeps decreasing, which is similar across the years. This shows a contrast result from the previous norms whereby men see children as a sign of wealth. Classical economists emphasized that ignoring the scale effects, the causal relationship between income and fertility is expected to be positive in the short run, and it could be negative in the long run [30]. In those days, men used their children to work on the farm which meant the more the number of children, the more labour they had to work with. Since farming is not the only and main source of wealth now, most men have to work to get their salary paid and then have to care for their children. Therefore, most men do not see children as a source of wealth. The result is contrary to what Zhang noted, that family income positively affects male fertility [31].

The community factors that influenced the ideal number of children between 2003 and 2018 are the region and community’s ideal number of children. The Northern region has much desire in many births compared to the Southern dwellers. Furthermore, men living in a community with ahigh ideal number of children have more desire than those living in a community with a low ideal number of children. Some types of religion favour large families and strongly oppose abortion, and some forms of birth control which the Northern dwellers follow Islam. In contrast, other community factors vary across the year of study. For example, in 2018, the Community level of education and community family planning are also significant. Men living in a community with high usage of family planning are less likely to desire high family size. Moreover, men living in a community with a high number of men that are educated with secondary schools or higher are less likely to desire a high number of children compared to those living in the community with a low number of educated men.

In conclusion, there is a need for more intervention to improve the following areas to control the population in Nigeria. They are in the area of improving the educational status, family planning, improving the standard of living, old age security, and modernization. Government should invest and make more efforts to improve the social amenities. Through modernization, the community will be developed, resulting in a lower birth rate because, from experience, modern societies now have a low birth rate.

## References

[1] Yeatman S., Sennott C., & Culpepper S. (2013). Young Women’s Dynamic Family Size Preferences in the Context of Transitioning Fertility. *Demography*, 50(5), 1715–1737. doi: 10.1007/s13524-013-0214-4 23619999 PMC3786023

[2] Hagewen K. J., & Morgan S. P. (2005). Intended and Ideal Family Size in the United States, 1970–2002. *Population Development Review*, 31(3), 507–527. doi: 10.1111/j.1728-4457.2005.00081.x 20376334 PMC2849141

[3] Yaya O. S., & Osanyintupin O. D. (2018). Determinants of Desired and Actual Number of Children and the Risk of having more than Two Children in Ghana and Nigeria. *Munich Personal RePEc Archive*.

[4] Makama G. A. (2013). Patriarchy And Gender Inequality In Nigeria: The Way Forward. *European Scientific Journal*, 9(17), 116–144.

[5] Dogo S. A. (2014). The Nigerian Patriarchy: When and How. *Cultural and Religious Studies*, 2(5), 263–275. doi: 10.17265/2328-2177/2014.05.002

[6] Odimegwu C., & Okemgbo C. N. (2008). Men’s Perceptions of Masculinities and Sexual Health Risks in Igboland, Nigeria. *International Journal of Men’s Health*, 7(1), 21–39

[7] Economic and Social Affairs. (2011). *Men in Families and Family Policy in a Changing World*. New York: United Nations.

[8] Aderinto A. A. (2017). Patriarchy and Culture: The Position of Women in a Rural Yoruba Community, Nigeria. *The Anthropologist*, 3, 2001(4), 225–230.

[9] Pinsker Joe (2019). Family, What Number of Kids Makes Parent Happiest. The Atlantic.com

[10] Stulp G, Barrett L (2016) Wealth, fertility and adaptive behaviour in industrial populations. *Philosophical Transactions of the Royal Society B*: *Biological Sciences* 2016, 371:20150153. 10.1098/rstb.2015.0153 PMC4822433 27022080

[11] Orbeta A. C Jr. (2005). Poverty, Vulnerability and Family Size: Evidence from the Philippines. *Asian Development Bank Institue Research Paper* *68*. Asian Development Bank Institue.

[12] Macro O. R. C. (2004). Demographic and health surveys. Calverton, MD: ICF Macro.

[13] Macro International. Institute for Resource Development. Demographic, Health Surveys, Nigeria. Federal Office of Statistics, & Nigeria. National Population Commission. (2008). Nigeria Demographic and Health Survey. IRD/Macro Incorporated.

[14] National Population Commission. (2013). *Nigeria demographic and health survey 2013*. National Population Commission, ICF International.

[15] National Population Commission (NPC) & ICF International: Nigeria Demographic and Health Survey 2018.Abuja, Nigeria, and Rockville, Maryland, USA: NPC and ICF 2019

[16] Bongaarts J (1978) A framework for analyzing the proximate determinants of fertility. *Population and development review*, 105–132.

[17] Bongaarts J (2015) Modeling the fertility impact of the proximate determinants: Time for a tune-up. *Demographic Research*, 33:535–560.

[18] Bongaarts J (1987) The proximate determinants of exceptionally high fertility. *Population and Development Review* 1987:133–139.

[19] Sear R, Lawson DW, Kaplan H, Shenk MK(2016) Understanding variation in human fertility: what can we learn from evolutionary demography?: The Royal Society; 2016. 10.1098/rstb.2015.0144 PMC4822424 27022071

[20] Betzig L(1997) People are animals. *Human nature*: *A critical reader*, 1–17.

[21] Kaplan H (1996) A theory of fertility and parental investment in traditional and modern human societies. *American Journal of Physical Anthropology*: The Official Publication of the American Association of Physical Anthropologists, 101:91–135.

[22] Shenk MK, Kaplan HS, Hooper PL(2016) Status competition, inequality, and fertility: implications for the demographic transition. Philosophical Transactions of the Royal Society B: *Biological Sciences*, 371:20150150. 10.1098/rstb.2015.0150 PMC4822430 27022077

[23] Von Rueden C (2014) The roots and fruits of social status in small-scale human societies. In *The psychology of social status*. Springer; 179–200

[24] Von Rueden C, Gurven M, Kaplan H (2011). Why do men seek status? Fitness payoffs to dominance and prestige. *Proceedings of the Royal Society B*: *Biological Sciences*, 278:2223–2232. 21147798 10.1098/rspb.2010.2145 PMC3107626

[25] Stulp G, Sear R, Barrett L (2016). The reproductive ecology of industrial societies, part I. *Human Nature*, 27:422–444. 27670436 10.1007/s12110-016-9269-4 PMC5107203

[26] Vining DR (1986) Social versus reproductive success: The central theoretical problem of human sociobiology. *Behavioural and Brain Sciences*, 9:167–187.

[27] Perusse D (1993). Cultural and reproductive success in industrial societies: Testing the relationship at the proximate and ultimate levels. *Behavioural and Brain Sciences* 16:267–283.

[28] Merlo J., Chaix B., Ohlsson H., Beckman A., Johnell K., Hjerpe P., et al. (2006). A brief conceptual tutorial of multilevel analysis in social epidemiology: using measures of clustering in multilevel logistic regression to investigate contextual phenomena. *Journal of Epidemiology & Community Health*, 60(4), 290–297. 16537344 10.1136/jech.2004.029454 PMC2566165

[29] Merlo J., Chaix B., Yang M., Lynch J., & Råstam L. (2005). A brief conceptual tutorial of multilevel analysis in social epidemiology: linking the statistical concept of clustering to the idea of contextual phenomenon. *Journal of Epidemiology & Community Health*, 59(6), 443–449. 15911637 10.1136/jech.2004.023473 PMC1757045

[30] Siddiqui R., (1996). The Impact of Socioeconomic Factors on Fertility Behaviour: A Cross-country Analysis. *The Pakistan Development Review*, Summer, 35(2), pp.107–128. 12321254

[31] Zhang L., (2011). Male Fertility Patterns and Determinants, Dordrecht, Springer.

