# Correction: Linkages between men’s wealth status and the ideal number of children: A trend and multilevel analysis of survey data in Nigeria

**DOI:** 10.1371/journal.pgph.0002093

**Published:** 2023-06-14

**Authors:** Ololade G. Adewole, Blessing I. Babalola, Kehinde O. Omotoso, Oyeyemi O. Oyelade, Elhakim A. Ibrahim

The first author’s name is spelled incorrectly. The correct name is: Ololade G. Adewole. The correct citation is: Adewol OG, Babalola BI, Omotoso KO, Oyelade OO, Ibrahim EA (2023) Linkages between men’s wealth status and the ideal number of children: A trend and multilevel analysis of survey data in Nigeria. PLOS Glob Public Health 3(3): e0001036. https://doi.org/10.1371/journal.pgph.0001036
